# A Narrative Review and Clinical Study on Er:YAG Laser Debonding of Ceramic and Composite Veneers

**DOI:** 10.3390/biomimetics10050295

**Published:** 2025-05-06

**Authors:** Jose Villalobos-Tinoco, Fabio Andretti, Clint Conner, Silvia Rojas-Rueda, Nicholas G. Fischer, Margiezel Pagan-Banchs, Carlos A. Jurado

**Affiliations:** 1 Department of Restorative Dentistry, Centro de Estudios Odontologicos (CEO), Queretaro 76050, Mexico; 2 Independent Researcher, Culiacan 80030, Mexico; 3 Division of Operative Dentistry, Department of General Dentistry, Health Science Center, College of Dentistry, The University of Tennessee, Memphis, TN 38163, USA; 4 Division of Biomaterials, Department of Clinical and Community Sciences, School of Dentistry, The University of Alabama at Birmingham, Birmingham, AL 35233, USA; 5 MDRCBB, Minnesota Dental Research Center for Biomaterials and Biomechanics, University of Minnesota, Minneapolis, MN 55455, USA; 6 School of Dental Medicine, Ponce Health Sciences University, Ponce 00716, Puerto Rico

**Keywords:** Er:YAG laser, composite resin veneers, ceramic veneers, minimally invasive dentistry, laser debonding

## Abstract

Background: Composite resin veneers have gained popularity due to their affordability and minimally invasive application as biomimetic restorations. However, long-term clinical challenges, such as discoloration, wear, and reduced fracture resistance, necessitate their replacement over time. Ceramic veneers, particularly feldspathic and lithium disilicate, offer superior esthetics and durability, as demonstrated by studies showing their high survival rates and enamel-preserving preparation designs. However, while ceramic veneers survive longer than composite resin veneers, ceramic veneers may need to be removed and replaced. Reports vary for using Er:YAG (erbium-doped yttrium aluminum garnet) lasers for the removal of existing veneers. Methods: A review was conducted to evaluate the effectiveness of removing restorative materials with an Er:YAG laser. A clinical study was included, highlighting the conservative removal of aged composite resin veneers using the Er:YAG laser. This method minimizes enamel damage and facilitates efficient debonding. Following laser application, minimally invasive tooth preparation was performed, and feldspathic porcelain veneers were bonded. Results: The review showed positive outcomes whenever the Er:YAG laser was used. In the case study, after a 3-year follow-up, the restorations exhibited optimal function and esthetics. Conclusions: Laser-assisted debonding provides a safe and predictable method for replacing failing composite veneers with ceramic alternatives, aligning with contemporary biomimetic principles.

## 1. Introduction

The integration of adhesive science with cosmetic dentistry is exemplified particularly well by composite resin veneers [1]. These veneers remain a favored option among many clinicians, especially when restoration is needed for just one or a few teeth—an approach often preferred in areas with limited access to advanced technologies or resources. Typical indications include correcting morphological abnormalities, concealing discolorations, and closing diastemas.

Over time, pH fluctuations, salivary enzymes, and humidity degrade its structure, leading to surface loss, cracks, and discoloration. Complete removal is often unnecessary, as repair preserves tooth structure and minimizes pulpal damage. Adhesion strength depends on surface preparation, composite viscosity, porosity, adhesive system, and restoration timing. A bonding agent facilitates chemical integration between new and existing composite layers [2].

Although composite resin veneers are gaining popularity due to their ability to meet biomimetic esthetic goals while preserving natural tooth structure [3,4], ceramic veneers remain the gold standard for anterior esthetic restorations [5,6]. This is largely because they offer consistently reliable results in terms of form, color, and mechanical properties. Veneers used to close diastemas or correct enamel defects demonstrate survival rates ranging from 74% to 96.3% over periods of 2 to 10 years. Such outcomes depend heavily on precise clinical protocols, careful material selection, and strict compliance with procedural guidelines. Over time, ceramic restorations have demonstrated outstanding success, particularly due to their long-term gloss retention, surface texture, and color stability [7].

Longitudinal outcomes can reach 100% survival rates for mechanical integrity, excellent scores for marginal adaptation, color stability, and excellent periodontal health, with probing depths ≤ 3 mm. It is paramount to address discoloration, gingival architecture, and tooth tissue preservation to successfully use porcelain veneers. For this purpose, a minimally invasive approach using porcelain veneers was planned in this case to preserve enamel, enhancing the bond strength and reducing marginal degradation [8].

Clinicians often face the challenge of replacing composite resin restorations while trying to preserve as much healthy tooth structure as possible, since excessive removal can reduce the tooth’s long-term durability [9,10]. As a result, the dental field is constantly exploring surface pretreatment techniques that can improve bonding effectiveness without damaging the restorative materials. One such method, sandblasting, can enhance adhesion by eliminating surface contaminants and revealing a clean substrate. However, it may also introduce surface and subsurface cracks in the material [11].

A study evaluating the accuracy of different auxiliary devices during the removal of direct composite resin veneers revealed that magnifying loupes enable superior visualization, with precise composite removal and fewer remnants, but also result in greater enamel/dentin loss due to over-preparation. This highlights the trade-off between the elimination of restoration remnants and tissue preservation. Auxiliary ultraviolet lighting did not significantly enhance outcomes compared to conventional methods, likely due to the composite’s fluorescence resembling natural tooth structure. Although electric motors can improve the removal technique, they still produce a trade-off as an unnecessary reduction in sound tooth structure still occurs [12].

The Er:YAG (erbium-doped yttrium aluminum garnet) laser offers several benefits for restoration removal, including its minimally invasive nature, allowing for the selective ablation of restorative materials while protecting healthy tooth structure. It also ensures thermal safety by keeping the rise in intrapulpal temperature below the critical 5.5 °C threshold, thereby avoiding potential pulp damage. Additionally, the laser provides efficient performance, with the ability to debond veneers in as little as 20 s, depending on their thickness, often surpassing the effectiveness of ultrasonic techniques [13].

The parameters for the removal of composite resin veneers are an adaptation from scenarios of the more well-published removal of ceramic veneers or the ablation of tooth hard tissues. Considering that there are limited reports showing the benefits of removing composite veneers with the Er:YAG laser, this article conducts a narrative review on the use of the Er:YAG laser as a safe, conservative alternative for the removal of composite resin restorations, with minimal side effects; this article highlights a case report presenting the steps for replacing composite resin veneers with porcelain veneers, using Er:YAG laser ablation, from treatment planning to a 3-year follow-up.

## 2. Materials and Methods

### 2.1. Literature Review

Articles published between January 2010 and January 2025 on the removal of restorations using an Er:YAG laser were searched in PubMed and Google Scholar. The following search strategy was used in PubMed: ((((Er:YAG laser) AND (composite resin veneers)) OR (ceramic veneers))) AND (laser debonding). The search in PubMed initially yielded a total of 31 articles. After an initial reading of the titles and the abstracts, seventeen articles were selected. Then, one article was excluded (a systematic review), and another article was excluded because it evaluated another type of laser, resulting in fifteen articles. For the Google Scholar search, the following keywords were used: “Er:YAG”, “Erbium Laser”, and “Debonding”.

As of January 2025, the Google Scholar search resulted in 174 articles. The following terms were used for the search: Er:YAG laser; composite resin veneers, ceramic veneers, minimally invasive dentistry; and laser debonding. After reading the titles and abstracts, one hundred and fifty-seven articles were excluded. From the seventeen selected articles, 3 articles were about other types of lasers, 1 article was a short communication, 1 reference was a book, 4 articles were about other subjects, 1 article used bovine teeth, and 1 article was repeated. This resulted in four articles selected. A further manual search was conducted on the references of the previous 20 articles, adding 7 more articles. The inclusion and exclusion criteria are listed in Table 1. Letters, books, book chapters, literature reviews, and articles with full text not available were excluded from this review. Only publications addressing the benefits of using an Er:YAG laser for the removal of resin composite veneers or other bonded restorative material were analyzed.

### 2.2. Case Report

**Figure 1:**
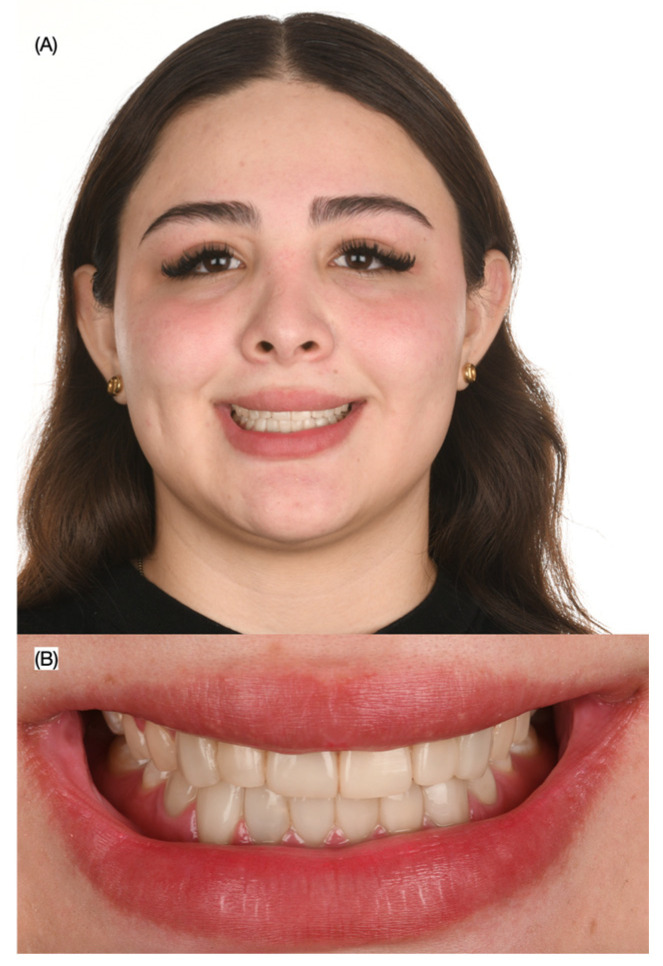
Initial extra-oral situation. (**A**) Face smiling and (**B**) close-up of the smile.

**Figure 2:**
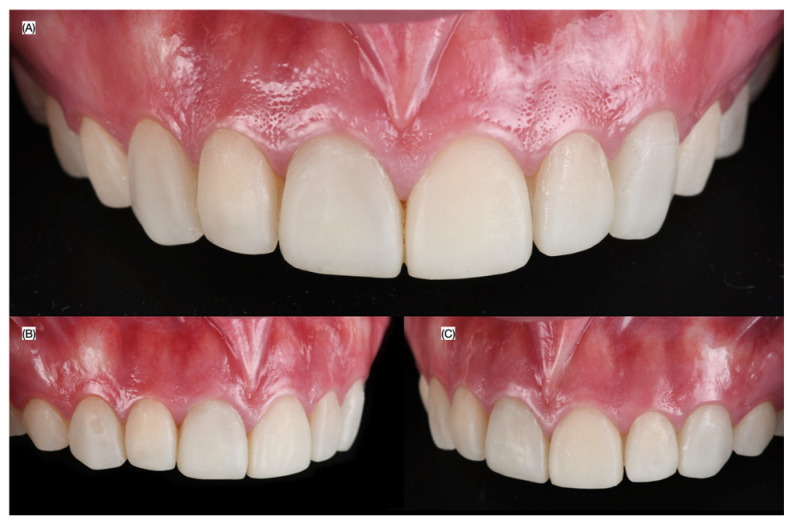
Initial intra-oral situation. (**A**) Frontal, (**B**) right, and (**C**) left side views.

A 30-year-old female patient presented to the clinic with the chief complaint of wanting to improve her smile. The patient was reported to have had direct resin composite veneers from the maxillary right first premolar to the maxillary left first premolar four years ago, but she dislikes the current situation as the restorations present some wear, and the initial anatomy provided (Figure 1 and Figure 2).

The patient was presented with a comprehensive treatment plan, including crown lengthening to improve the gingival architecture of the anterior teeth, followed by feldspathic porcelain veneers from the maxillary right first premolar to the left first premolar. However, the patient declined the crown lengthening procedure and requested only the ceramic veneers. The patient was informed that, due to the low smile line, avoiding the crown lengthening procedure was still an acceptable option. Additionally, the patient was informed about the option of having the resin composite veneers removed in a minimally invasive manner with an Er:YAG laser, instead of using traditional dental burs, and she accepted this option.

The patient was informed that a diagnostic wax-up would be completed, followed by an intra-oral mock-up to evaluate the proposed restorations. Once the diagnostic wax-up (Wax GEO Classic, Renfert, Hilzingen, Germany) and mock-up (Integrity, Dentsply Sirona, Charlotte, NC, USA) were performed, the patient was satisfied and pleased with the esthetic outcome and consented to proceed with treatment for feldspathic veneers from the maxillary right first premolar to the left first premolar.

**Figure 3:**
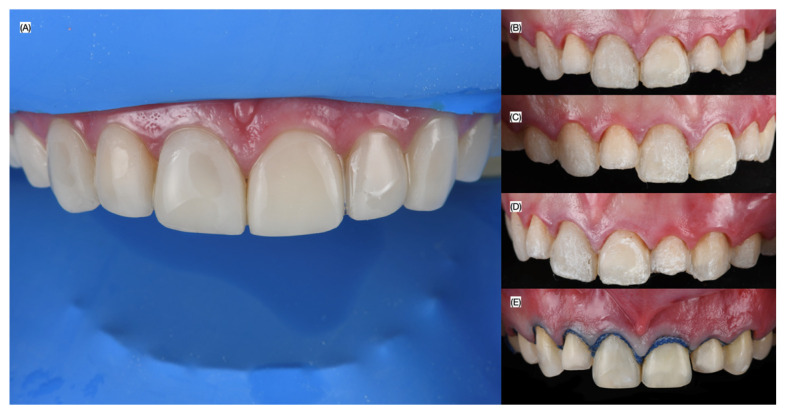
Removal of composite veneers. (**A**) Placement of modified dental dam; (**B**) frontal, (**C**) right side, and (**D**) left side views; (**E**) cord packed prior impression.

At the next visit, isolation of the maxillary arch from the right first molar to the left first molar was achieved using a dental dam (Dental Dam, Nic Tone, Bucharest, Romania), stabilized with clamps (#00 Clamp, Hu-Friedy, Chicago, IL, USA). The existing resin composite veneers were removed with an Er:YAG laser, and veneer preparations were refined using a dedicated veneer preparation bur kit (Solution Laminate Veneer Preparation System, Brasseler, Savannah, GA, USA). A double retraction cord technique was employed using Ultrapak #000 (Ultradent, South Jordan, UT, USA) (Figure 3) for further isolation and visibility.

Final tooth preparations were polished using polishing discs (Sof-Lex XT Disc, 3M, St. Paul, MN, USA) in a sequence of coarse, medium, and fine grits. A final digital impression (Aoralscan 3, Shinning 3D, Hangzhou, China) was then taken for the maxilla, mandible, and both arches in occlusion. The final veneer restorations were digitally designed (Dental-CAD 3.1, Exocad, Darmstadt, Germany) following the contours previously approved in the diagnostic mock-up.

Next, the ceramic laminate veneer restorations were milled from lithium disilicate (shade A1, Amber Mill, Hass Bio, Gangneung, South Korea). The restorations were tested to evaluate their margins, contour, and shade. The patient approved them as shown and asked to proceed with the final cementation process.

The final ceramic veneers were first treated with 5% hydrofluoric acid (IPS Ceramic Etching Gel, Ivoclar, Schaan, Liechtenstein) for 20 s, followed by cleaning in an ultrasonic bath with 96% isopropyl alcohol for 5 min. Silane (Monobond Plus, Ivoclar, Schaan, Liechtenstein) was then applied for 60 s. The teeth were sandblasted with water and 20 µm aluminum oxide particles, followed by the application of 37% phosphoric acid (Total Etch, Ivoclar, Schaan, Liechtenstein) for 20 s. The teeth were rinsed, dried, and primed before applying adhesive (Optibond FL, Kerr, Brea, CA, USA).

**Figure 4:**
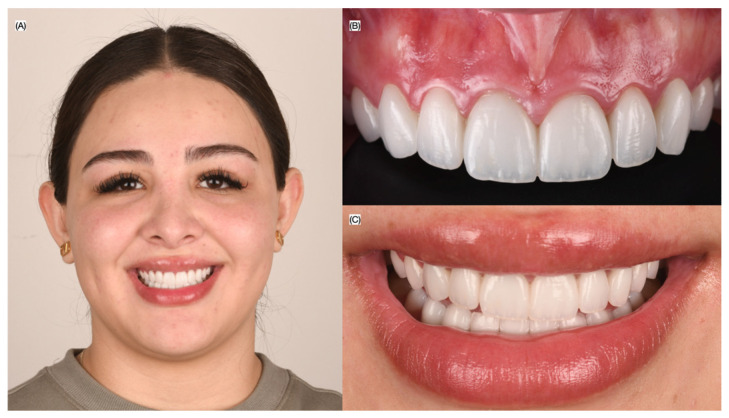
Final bonded ceramic veneers. (**A**) Patient smiling, (**B**) intra-oral view, and (**C**) close-up of the smile.

Finally, the veneers were cemented with light-cured luting resin cement (Choice 2, Bisco Inc., Schaumburg, IL, USA) under rubber dam isolation, starting with the two central incisors, followed by the laterals, canines, and first premolars. The patient was satisfied with the contour and shade of the veneer restorations (Figure 4).

**Figure 5:**
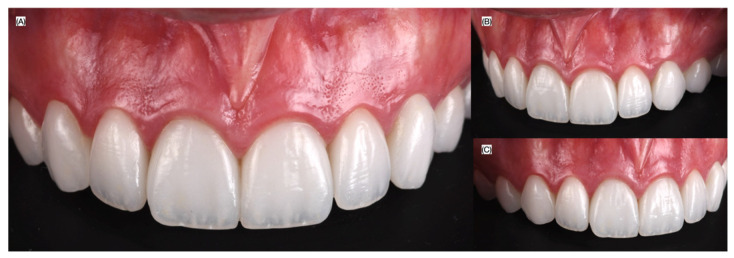
Three-year follow-up. (**A**) Frontal, (**B**) left, and (**C**) right side intra-oral views.

The patient was advised to brush her teeth three times a day and was instructed to attend follow-up visits every 6 months to evaluate the restorations and receive dental prophylaxis. Additionally, the patient was provided with an occlusal guard to protect the restorations at night. At the 3-year follow-up visit, the patient remained satisfied with the shade and shape of the laminate veneers (Figure 5).

A summary of the clinical procedures performed can be seen in Table 2.

## 3. Results

### 3.1. Literature Review Outcomes

The literature offers several studies evaluating the effectiveness of using Er:YAG to remove bonded materials on teeth. The methodological quality of each study was assessed by one reviewer (F.A.). The findings from a brief literature review of articles detailing the outcomes are presented in Table 3.

The following aspects were analyzed for the articles on the removal of veneers using an Er:YAG laser: specimen randomization, control group, standardized specimens, manufacturer’s instructions, single operator, availability of outcome data, and overall assessment (Table 4).

### 3.2. Clinical Study Outcomes

The workflow implemented in this clinical study successfully met the patient’s esthetic demands. The removal of the veneers using the Er:YAG laser provided a more conservative approach and significantly expedited the process compared to traditional methods of restoration removal performed manually with a high-speed handpiece. The periodontal soft tissues surrounding the restorations remained undamaged during the removal process, and their health was preserved both during the procedure and after the cementation of the veneer restorations.

**Figure 6:**
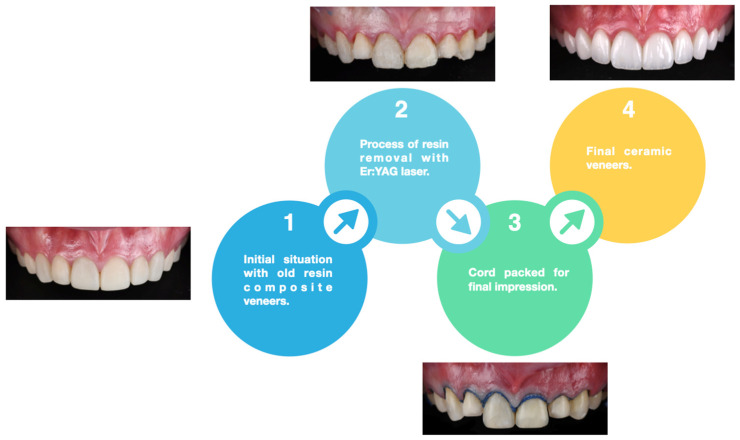
Summary of the clinical study workflow.

The patient was provided with an occlusal guard to protect the restorations at night. Additionally, they received oral hygiene instructions and were advised to undergo dental prophylaxis every six months. During these follow-up visits, the restorations were re-evaluated to ensure their continued success. The clinical workflow performed in this study is illustrated in Figure 6.

## 4. Discussion

Although composite resin veneers offer esthetic benefits, their longevity is generally limited to around five years [8]. For this reason, the authors of this case chose to recommend replacement with porcelain veneers. Er:YAG lasers offer a precise method for selectively removing composite resin from enamel surfaces, especially in permanent teeth. This precision is due to water-mediated photomechanical interactions, which differ significantly from the more invasive action of dental burs that tend to remove healthy tooth structure along with the restoration. Enamel treated with Er:YAG lasers develops a rough, microretentive surface featuring exposed enamel rods, closely resembling the ideal pattern produced by acid etching for adhesive bonding. These laser-treated surfaces display features such as open tubules and roughened enamel, which promote strong adhesive bonds. Additionally, the lack of carbonization or thermal damage confirms the safety and appropriateness of the Er:YAG laser settings used (4 W power with water cooling). Lasers may reduce iatrogenic damage, but improper higher pulse rates (25 Hz) bring the risk of excessive heating and unintentional tissue ablation, which stresses the importance of operator expertise [25].

In addition to vaporizing the composite resin, a 2940 nm wavelength Er:YAG laser selectively vaporizes intertubular dentin (higher water content) while preserving peritubular dentin. This mechanism creates a microroughened surface ideal for adhesion without compromising pulp safety [25]. The comparison of ablation rates across composite resins (microfilled, hybrid, condensable) and dental hard tissues (primary/permanent enamel and dentin) revealed that the composite resin ablation rates were 5–10× higher than enamel, enabling preferential resin removal while preserving intact enamel. Hybrid composite resins showed the most favorable ablation rates compared to enamel and demonstrated optimal differential ablation compared to microfilled (prone to rough ablation) and condensable (fiberglass-reinforced) resins [26].

In this case report, the tooth preparation was limited to smoothing line angles and establishing an insertion path. This approach boosts the conservative protocol through diagnostic validation and guided preparation, controlled moisture conditions during the bonding procedures by using a rubber dam, and minimal thickness requirements for the felspathic material, aligning with enamel preservation and biomimetic goals [27].

The use of Er:YAG laser conditioning is an alternative to conventional acid etching for preparing tooth surfaces prior to Class V restorations, and pit and fissure sealants were investigated [28]. There were significant differences in microleakage between occlusal (enamel) and cervical (cementum/dentin) margins. In Class V restorations, the group using laser conditioning on cementum with composite restoration exhibited higher microleakage compared to groups treated with acid etching or combined methods. However, further research—with larger sample sizes and standardized protocols—is needed to validate these in vitro findings and to determine the clinical efficacy of laser etching in reducing microleakage.

A qualitative comparison of the effectiveness of Er:YAG lasers revealed that this technology performed better than the conventional technique for removing composite remnants but produced more enamel ablation [14]. A tungsten carbide bur and enamel ablation (two Er:YAG laser intervals) were used after bracket debonding from 12 extracted premolars (four premolars served as a control). The Er:YAG laser removed composite remnants faster, applied for 10 s, compared to the variable time with the tungsten carbide burs. The Er:YAG laser is a viable method for removing composite remnants after bracket debonding, performing better than mechanical removal. The amount of enamel ablation is a disadvantage, and further studies with lower energy intervals and pulse repetitions are recommended to find ideal specifications for Er:YAG laser application without affecting enamel.

The findings from a study on the use of Er:YAG lasers for debonding orthodontic brackets, focusing on their effectiveness, safety concerning enamel damage, and recommendations for future monitoring of enamel health revealed that the laser application facilitates removal of brackets, particularly ceramic ones, while significantly reducing the amount of adhesive residue left on the enamel surface [15]. The highlighted superior debonding efficiency of Er:YAG lasers for bracket debonding compared to traditional mechanical methods is encouraging, but it is essential to consider long-term enamel health. Regular follow-up appointments should include evaluations of enamel integrity and the presence of any residual adhesive that may contribute to caries risk.

The effectiveness and safety of Er:YAG lasers for the debonding of lithium disilicate crowns were investigated, with particular attention to whether laser power settings influenced the procedure when applied to crowns of varying thicknesses [16]. A laser power setting of 5 W was found to be optimal for debonding 1 mm thick lithium disilicate crowns, demonstrating both safety and efficacy for this specific thickness. In contrast, higher power settings of 5.6 W and 5.9 W, tested on thicker crowns (1.5 mm and mixed thickness), resulted in reduced debonding times but raised concerns regarding dental pulp safety. Although increased laser energy may improve efficiency, it also poses risks, particularly for thinner crowns. Therefore, clinicians should prioritize minimizing heat transmission to protect the underlying vital tissues. By following the recommended power settings—5 W for 1.0 mm crowns and cautiously increasing output for thicker ones—dentists can enhance the debonding process while reducing potential harm to pulp vitality.

One of the most significant aspects is the laser tip’s distance from the surface. It has been found marked differences in debonding times between non-contact mode (NCM) and contact mode (CM) when using Er:YAG lasers—12.6 s for NCM compared to 96.3 s for CM [17]. This notable reduction in debonding time underscores the potential of NCM to improve procedural efficiency in clinical practice by decreasing chair time and allowing for faster patient turnover. While the enhanced efficiency of NCM is evident, it is essential to consider safety aspects, particularly the rise in dental pulp temperature during debonding. This study reported an average temperature increase of 4.2 °C with NCM and 2.9 °C with CM. Although both values remained below the critical threshold of 5.25 °C for pulp survival, the greater temperature rise associated with NCM suggests a heightened risk of thermal injury if not closely monitored. Therefore, clinical decision-making must balance the efficiency benefits of NCM with the potential for pulp tissue damage. The evaluation of various laser settings—such as 360 mJ at 15 Hz and 400 mJ at 10 Hz—revealed a notable impact on both debonding duration and temperature changes in the pulp, emphasizing the importance of selecting and optimizing parameters to ensure both efficacy and safety [17].

Considering these findings, several strategies have been proposed for improving the clinical application of Er:YAG lasers in crown debonding. Primarily, practitioners are encouraged to utilize NCM for its time-saving advantages if pulp temperature is carefully monitored throughout the procedure. Furthermore, ongoing research is recommended to investigate a wider range of laser settings, with the aim of identifying combinations that effectively balance performance and thermal safety. Lastly, emphasis should be placed on continued education and hands-on training in laser technology, ensuring that dental professionals are well prepared to use these tools with competence and confidence [17].

The Er:YAG laser has become an important tool in modern dental practice, especially for the removal of all-ceramic restorations [18]. To ensure optimal outcomes for both the tooth structure and ceramic materials, clinicians must follow specific preparation protocols before initiating the debonding process. These include thorough tooth surface preparation, where enamel and dentin are appropriately reduced to establish a suitable bonding substrate for future restorations. Ceramic material selection is equally crucial; materials such as leucite or lithium disilicate should be properly bonded using a dual-cure resin cement to ensure durable adhesion. In addition, clinicians must set the laser parameters carefully, typically using a 2940 nm wavelength, around 3 W of power, a pulse duration, and a frequency of approximately 10 Hz (e.g., 300 mJ), tailored to the specific clinical context. An air–water cooling mechanism should also be employed to minimize thermal impact on both dental and ceramic structures, thereby preserving the integrity of the restoration and surrounding tissues.

During the debonding procedure, several considerations should be considered to maximize the benefits of the Er:YAG laser [18]. In terms of effective debonding, the laser successfully removes resin-luted ceramic restorations without damaging either the tooth surface or the restoration itself, preserving the possibility for future rebonding. The laser functions through controlled mechanisms, primarily thermal and photoablation, which maintain resin and dentin temperatures within safe physiological limits, thereby minimizing the risk of heat-induced damage. Regarding bond strength, while the use of the Er:YAG laser may result in a slight reduction in shear bond strength compared to untreated controls, it does not significantly compromise the rebonding strength of the restorations. These findings support Er:YAG laser clinical applicability for procedures requiring restoration, replacement, or reattachment [23].

The influence of pulse duration and cooling ratios on the debonding process and their thermal impact on the dental pulp was previously evaluated, revealing that shorter pulse durations of 50 μs and 100 μs significantly decreased debonding times compared to the longer 300 μs setting [24]. This highlights the clinical efficiency of shorter pulse durations in achieving faster porcelain laminate veneer removal, making them preferable in practice. While the water-to-air (W/A) cooling ratio had minimal impact on debonding time and pulp temperature at shorter pulse durations, it became more relevant with extended durations. Notably, the highest rise in pulp temperature—3.4 °C—was observed with the 300 μs setting at a 1:1 W/A ratio. In contrast, the 50 μs and 100 μs time maintained pulp temperatures below the critical threshold of 5.5 °C, thereby improving procedural safety. Based on these findings, the use of 50 μs or 100 μs pulse durations is recommended for the safe and efficient debonding of porcelain laminate veneers, as they reduce procedure time while keeping dental pulp temperature within safe physiological limits, minimizing the risk to tooth structures [24].

The efficiency of the debonding process is greatly affected by the thickness of ceramic veneers, with thinner veneers (0.5 mm) enabling better transmission of laser energy. As a result, they can be removed more quickly and with less residual adhesive left on their surface compared to thicker veneers (1.0 mm) [25]. Veneers measuring 1.0 mm not only required more time to debond but also led to a greater increase in temperature. In contrast, 0.5 mm veneers were successfully removed in under six seconds with minimal heat generation. These results emphasize the need to consider veneer thickness when adjusting laser power settings to ensure effective and safe clinical outcomes [35]. The authors of a study on the use of an Er:YAG laser to debond porcelain veneers without damaging the underlying tooth and retaining the veneer examined the transfer of energy through veneers with different thicknesses. They found that Er:YAG laser irradiation efficiently debonds porcelain veneers while preserving tooth structure. Maintaining veneer integrity throughout this process depends on the porcelain’s flexural strength. Using FTIR spectroscopy and visual inspection, testing the absorption characteristics and ablation threshold of the veneer cement was a key component of the methodology. This showed that while the bonding cement had a broad H_2_O/OH absorption band, the veneer materials showed no water absorption bands. The findings also showed that while lithium disilicate veneers transmitted approximately 26.5–43.7% at comparable thicknesses, leucite ceramic veneers transmitted about 11.5–21% of the laser energy. Using the Er:YAG laser in an average period of 113 s, leucite veneers were entirely removed; generally, these results imply that veneer flexural strength, rather than water uptake, is the main determinant of veneer fracture during laser debonding [21].

A study evaluating the debonding strength of laminate veneers after Er:YAG laser application on each veneer for 9 s using a scanning method reported significantly higher shear bond strengths (27.28 ± 2.24 MPa) in the control group compared to the laser-irradiated group (3.44 ± 0.69 MPa). This showed that laser energy can degrade the adhesive resin and substantially reduce the shear bond strength of laminate veneers. Er:YAG laser application thereby efficiently reduces the shear bond strength of laminate veneers, therefore enabling removal [21].

The use of Er:YAG lasers for veneer removal has been extensively studied, with particular focus on key elements such as debonding efficiency, tooth structure preservation, and clinical relevance [22]. One of the most critical factors influencing successful laser-assisted debonding is irradiation time. While the total time required for veneer removal typically stays under four minutes, it is essential to differentiate between actual working time and irradiation time, particularly when operating with pulsed laser systems. Variables like pulse duration and the time taken to reach peak power can have a notable impact on the procedure’s effectiveness. However, further investigation is needed to fully understand their precise influence on the debonding process [22].

Significant differences have been identified in the energy transmission properties of various crown materials, which directly impact the effectiveness of laser-assisted debonding procedures. E.max CAD crowns demonstrated superior laser energy transmission, enabling a more efficient debonding process [23]. In contrast, ZirCAD crowns were found to transmit approximately 80% less laser energy, resulting in a more time-consuming and technically challenging removal. Due to their thicker structure, ZirCAD crowns require higher laser energy settings to adequately reach and degrade the bonding cement, especially in hard-to-access areas such as contact points. This marked contrast in energy transmission capabilities underscores the critical importance of careful material selection in clinical scenarios involving laser debonding [23].

Erbium lasers have demonstrated a high success rate exceeding 95% for the intact removal of ceramic prostheses, markedly outperforming conventional rotary instruments [24]. This laser-assisted technique offers the advantage of preserving underlying structures, with no significant surface or chemical alterations detected on natural teeth or implant abutments. The effectiveness of erbium lasers is evident in the average removal times for various ceramic restorations: veneers take around 2.25 min, crowns approximately 6.89 min, and fixed partial dentures about 25 min per abutment. Although high-speed burs can remove composites more quickly (63.1 s) than lasers (121 s at 20 Hz), they tend to affect a larger area, whereas lasers provide a more conservative approach (0.800 mm vs. 0.372 mm of tooth structure at 20 Hz, respectively) [25]. Additionally, lithium disilicate crowns are typically easier and quicker to remove than zirconia crowns, which require longer treatment times. This procedural efficiency enhances patient comfort and contributes to a more streamlined, time-saving clinical workflow [25].

Aksakalli et al. conducted a comparison between two widely used surface treatment techniques—Er:YAG laser etching and hydrofluoric acid (HFA) etching—evaluating their effectiveness in achieving bond strength and their safety with respect to potential damage to porcelain surfaces and surrounding oral tissues [26]. HFA etching demonstrated the highest shear bond strength, averaging 10.8 ± 3.8 MPa, making it the most effective option for bonding orthodontic brackets to porcelain surfaces. In comparison, Er:YAG laser etching produced bond strengths that, while lower, remained within clinically acceptable limits for orthodontic applications. Considering the critical role of reliable bonding in orthodontic treatment, clinicians are encouraged to weigh both bond strength and safety when selecting an etching method. This study supports a strong recommendation for the clinical use of Er:YAG laser etching, particularly when safety is a primary concern [26].

Juntavee et al. investigated the effectiveness of various surface treatments, focusing on acid etching and Er:YAG laser treatment, in terms of their impact on the shear bond strength of ceramic brackets bonded to materials such as porcelain fused to metal, IPS Empress CAD, and IPS e.max CAD [27]. The findings revealed that Er:YAG laser treatment achieved bond strength comparable to that of a 15 s acid etching protocol, establishing it as a viable alternative. Importantly, Er:YAG laser treatment also reduces the risk of damaging the ceramic surface during the debonding process, which presents a notable clinical advantage [27].

Ismatullaev et al. compared the efficacy of laser etching and acid etching on bond strength, with particular attention to enamel and dentin surfaces, offering recommendations for clinical application based on current evidence [28]. The morphological changes caused by each technique significantly influence bonding performance. Laser etching generates a unique surface characterized by demineralization and the opening of dentinal tubules without forming a smear layer, thereby enhancing adhesive penetration into exposed collagen fibrils. Conversely, acid etching typically results in a generalized roughening of the enamel surface, which may not consistently support optimal bonding. These distinct surface alterations affect how well adhesive systems perform when paired with either method, emphasizing the importance of choosing an appropriate etching technique for clinical success [28].

Based on these findings, the following recommendations are proposed for clinical practice [29,36,37,38,39]:

o Laser etching, specifically with the Er:YAG scanning handpiece, may be an effective alternative to traditional acid etching for both enamel and dentin. This approach yields a more uniform surface morphology, which may enhance bond quality.
o Clinicians should adjust laser parameters, such as 120 mJ, 10 Hz, and 1.2 W, to improve bond strength. The application of double irradiation to dentin may increase the adhesive surface area and improve bond strength values.
o It is essential to assess the variability in bond strength outcomes associated with various etching methods. Clinicians must evaluate material-specific and procedural factors to obtain reliable results.
o Further clinical studies are necessary to enhance the understanding of the comparative effects of laser etching and acid etching across various treatment contexts.
o Observe alterations in dentin tubules: The impact of etching on the diameter of dentin tubules warrants consideration, as reduced diameters may hinder resin infiltration and influence bonding effectiveness. Understanding these effects facilitates more informed decisions in the selection of etching techniques.

Emphasizing the need for clinician training and knowledge with the laser system, Oztoprak et al. provided evidence-based advice for the safe and efficient use of Er:YAG lasers in the debonding of porcelain laminate veneers [29]. Efficient veneer removal depends on precise knowledge of certain laser parameters, including power and wavelength, and expertise in scanning methods. To preserve best practices in laser-assisted debonding [29], clinicians are urged to remain current with the literature and engage in hands-on training. Routine assessment of bond strength is also recommended to gauge the efficacy of the treatment, hence guiding practitioners to change their strategy depending on clinical results and patient input. The safety and efficiency of Er:YAG laser use in patient care is supported by this constant review [29].

Considering that this review combines in vitro, ex vivo, and clinical studies, care should be taken not to extrapolate the findings to similar clinical scenarios, especially considering the methodological variability involved in the studies. Further research should validate these findings using similar materials and assess long-term effects on bonding; further research should focus on parameter optimization, i.e., refining pulse durations (e.g., ultra-short pulses) to reduce thermal stress; the use of adjunctive technologies, combining air abrasion or enzymatic solutions to enhance adhesive breakdown; and the development of compact, cost-effective Er:YAG handpieces for chairside debonding procedures.

## 5. Conclusions

The Er:YAG laser creates clean, microretentive surfaces on dentin and enamel, making it a valuable tool for conservative dental treatments. With improvements in laser settings and user training, its precision and range of use are likely to grow. This technology marks a step forward in restorative dentistry by offering accuracy and tooth preservation that traditional methods cannot match. Although some material-related challenges remain, ongoing progress in laser technology and surface treatments is expected to increase its usefulness, especially for minimally invasive procedures. Er:YAG laser-assisted veneer removal is a safe and reliable option for replacing old composite veneers with feldspathic porcelain ones, supporting modern, tooth-conserving approaches to restorations.

## Figures and Tables

**Table 1:** Inclusion and exclusion criteria of the search performed.

Criterion	Inclusion	Exclusion
Time period	Publications available between January 2010 and January 2025	All publications published before January 2010
Language	English	Non-English
Type of articles	All research types, including primary research (e.g., experimental studies, clinical trials, and pilot studies). Full text available.	Letters, books, book chapters, and full text not available

**Table 2:** Summary of the clinical workflow performed.

Step	Description
Patient Complaint	Dissatisfaction with worn resin composite veneers on maxillary premolars and anterior teeth.
Proposed Treatment	Feldspathic veneers with optional crown lengthening; patient chose veneers only.
Initial Steps	Diagnostic wax-up and intra-oral mock-up approved by the patient.
Resin Removal	Resin veneers removed using Er:YAG laser; preparations refined with veneer bur kit.
Tooth Preparation	Teeth polished and digitally scanned for final veneer design and fabrication.
Veneer Cementation	Veneers cemented with light-cured resin cement under rubber dam isolation.
Post-Treatment Care	Provided oral hygiene instructions, occlusal guard, and biannual follow-up schedule.
Outcome	Patient satisfied at 3-year follow-up with the veneers’ shade, shape, and functionality.

**Table 3:** Publications on clinical efficacy of using Er:YAG to remove bonded restorative materials.

Author/Date	Groups	Laser	Methods	Results/Conclusion
Luong & Shayegan (2018) [14]	Seventy-two extracted third molars. Class V cavities with occlusal margins in enamel and cervical margins in cementum/dentin. Restored either with composite resin or resin-modified glass ionomer cement. Three surface conditioning methods: conventional acid etching, Er:YAG laser etching, and a combination of acid etching and laser ablation. Groups were formed by the position of the cavity (buccal or lingual), substrate (enamel or cementum), and protocol (conventional acid etching, laser, and association of etching and laser).	Er:YAG laser (Fotona) in Quantum Square Pulse (QPS) mode (1.2 W, 10 Hz, wavelength 2.94 μm)	Sixty teeth (10 per group). 2% methylene blue dye solution for 24 h. The samples were sectioned in the buccolingual direction, using a water-cooled diamond saw (Leitz 1600 saw microtome; Ernst Leitz Wetzlar GmbH, Wetzlar, Germany). All sections were viewed under stereomicroscope.	The application of the Er:YAG laser, beneath the resin composite, the resin-modified GIs, and the fissure sealant placement, may be an alternative enamel and dentin etching method to acid etching; however, further research with larger samples and recognized consensus standards is needed.
Almeida et al. (2009) [15]	Twelve extracted premolars and composite remnants were removed by tungsten carbide bur and 2 Er:YAG laser intervals, and four premolars served as a control.	Er:YAG laser at 100 mJ, and Er:YAG laser at 120 mJ	Teeth were randomly divided into three groups: composite removal with a tungsten carbide bur, Er:YAG laser at 100 mJ, and Er:YAG laser at 120 mJ. Enamel sections were prepared for scanning electron microscopy (SEM) analysis, and digital images were analyzed by three calibrated dentists for composite remnants and enamel ablation using a ranking system.	Er:YAG laser (both 100 mJ and 120 mJ) removed composite remnants more effectively than the tungsten carbide bur method (*p* < 0.05). Er:YAG methods caused significantly more enamel damage compared to the tungsten carbide bur method. The Er:YAG laser is useful in removing composite remnants after bracket debonding, but may cause significantly higher enamel damage than conventional methods.
Dostalova et al. (2016) [16]	Forty ceramic and metal brackets (Clarity™ Advanced and Victory Series™; 3M Unitek, Monrovia, CA, USA) were standardly bonded to buccal polished enamel surfaces of 30 caries-free human third molars.	Er:YAG laser (FJFI CVUT) 280 mJ, 250 µs long, repetition rate 6 Hz, spot focus 1 mm, and 140 s	Two types of adhesive resins (Transbond™ XT Light Cure Adhesive; 3M, and Variolink II; lvoclar). Before debonding, the brackets in the laser group were irradiated. The control group was debonded without the laser irradiation. During the bracket irradiation, temperature changes inside the tooth were monitored using a thermal image infrared camera. The enamel surface was investigated by SEM.	Er:YAG laser-treated surfaces exhibited minimal damage and were cleaner than those subjected to mechanical removal methods.
Gozneli et al. (2023) [17]	Twenty-seven intact premolars were prepared to fabricate lithium disilicate CAD/CAM full-coverage crowns in three different thicknesses: 1.0, 1.5 mm, and mixed thickness (n = 9). Each thickness group was divided into 3 subgroups and subjected to Er:YAG laser at different wattages (5.0, 5.6, and 5.9 W) to determine the appropriate wattage for each thickness. The removal time and temperature rise values were recorded. Kruskal–Wallis test was performed to evaluate any significant differences in removal time, Mann–Whitney U test with Bonferroni correction for multiple comparisons, and Pearson chi-square test for temperature rise over the critical value (*p* < 0.05).	Er:YAG laser (Fidelis III; Fotona)	Prepared tooth surfaces were scanned (inEos X5 device (Dentsply Sirona), and the crowns were designed (inLab 18.1, Dentsply Sirona) with 1.0 mm, 1.5 mm, and mixed thicknesses (marginal third: 1.0 mm, middle third: 1.5 mm and occlusal third: 2 mm) (n = 9). IPS E.max CAD blocks (MT, A1; Ivoclar Vivadent, Schaan, Liechtenstein) were milled (inLab MC X5, Dentsply Sirona). After try-in of the as-milled lithium disilicate crowns, the crystallization was performed (Programat P310; Ivoclar Vivadent, Liechtenstein).TT: 3STE Crown intaglio surfaces: 4% HF gel (IPS Ceramic Etching Gel; Ivoclar, Liechtenstein) for 20 s; rinsing and drying for 30 s. Silane (Monobond S; Ivoclar Vivadent, Liechtenstein) active application for 60 s. Laser application: scanning method, zigzag pattern, 7–8 mm distance, initially at the buccal surface, up and down from the incisal margins to the cervical margins for 30 s. Application pattern repeated on the palatal surface for 30 s, then buccal and palatal line angles/cusps for 30 s; 15 s for buccal line angles/cusps, and 15 s for palatal line angles/cusps; then applied to the occlusal surface, mesially to distally for 30 s. Finally, laser was applied to the interproximal areas from the lingual and buccal sides for 30 s. Total laser application period: 2 min 30 s for the first laser application. Crown removal was attempted. When no movement was detected, the laser application was repeated at half of the first duration at each application surface. The total laser application period was limited to 15 min for each sample.	Power setting of 5 W is optimal for debonding 1 mm thick lithium disilicate crowns. Higher power settings of 5.6 W and 5.9 W were tested for thicker crowns (1.5 mm and mixed thickness), which resulted in shorter debonding times but raised concerns regarding dental pulp safety. Recommended power settings: 5 W for 1.0 mm crowns and cautiously increasing power for thicker crowns.
ALBalkhi et al. (2018) [18]	Forty extracted non-carious human maxillary premolars were prepared to receive porcelain laminate veneers. Sixteen of them were divided into two groups, each of which comprised eight samples based on the application mode: group A with non-contact mode (NCM), and group B with contact mode (CM).		Laser parameters (360 mJ, 15 Hz). Loading: 15 N force on specially fabricated veneer cervical margins.	NCM was more efficient. Additional groups of the same mode and number of samples were tested with different laser parameters of energy and frequency: group C (400 mJ, 10 Hz), group D (270 mJ, 15 Hz), and group E (300 mJ, 10 Hz). Failure mode was determined and classified.
Karagoz-Yldirak & Gozneli (2020) [19]	Thirty-six extracted human maxillary premolars. Six groups based on varying pulse durations (50 μs, 100 μs, and 300 μs) and W/A cooling ratios (1:1 and 3:3).	Er:YAG laser (Fidelis III; Fotona)	Laser power of 3 W, (frequency 10 Hz, pulse energy 300 mJ) with 2940 nm wavelength, 100 μm pulse duration for 9 s. The non-contact mode (focused mode) handpiece (R02), 0.9 mm spot diameter, 0.0064 cm^2^ spot area at disc, 7–8 mm distance, 472 W/cm^2^ average power density.	Shorter pulse durations of 50 μs and 100 μs significantly reduced debonding times (DTs) compared to the longer duration of 300. DTs for the shorter pulses ranged from 7.4 to 17 s, while the 300 μs pulse duration resulted in a prolonged DT of 104 s. This finding underscores the efficiency of shorter pulse durations in facilitating quicker debonding of PLVs, making them the preferred choice for clinical applications. Optimal laser parameters for the safe and effective debonding of porcelain laminate veneers involve the use of pulse durations of 50 μs or 100 μs.
El-Damanhoury et al. (2022) [20]	Forty-eight maxillary central incisors restored with flat lithium disilicate veneers.	Er:YAG laser (Fidelis AT; Fotona); wavelength 2940 nm; emission mode: Very short pulse mode; pulse duration: 100 μs; delivery system: 7-mirror; articulated arm with non-contact handpiece (R02); energy distribution: inhomogeneous; average power: 1.5 W (150 mJ × 10 Hz), 3.0 W (300 mJ × 10 Hz), and 5.4 W (360 mJ × 10 Hz); spot diameter at the tissue: 0.9 mm; average power density at the tissue: 235.79 W/cm^2^; 471.57 W/cm^2^; 848.83 W/cm^2^; water irrigation: 40 mL/mm; air and aspirating airflow: 40 mL/mm	The labial enamel of 48 maxillary central incisors was flattened and polished. The teeth were restored with flat lithium disilicate ceramic veneers (4.0 mm × 6.0 mm) with one of two different thicknesses (0.5 and 1.0 mm). Veneer debonding was performed with an Er:YAG laser with a wavelength of 2940 nm, pulse duration of 100 μm (VSP mode), 10 Hz, and one of the three laser power settings: 1.5 W (150 mJ), 3.0 W (300 mJ), and 5.4 W (540 mJ) (n = 8). Veneer detachment time and intra-pulp temperature change (ΔT) were measured. Statistical analysis was performed using the two-way ANOVA and Bonferroni’s post hoc test (α = 0.05). The correlation between debonding time and temperature change was calculated using Pearson’s correlation.	Laser power setting of 5.4 W effectively decreased the time required for debonding lithium disilicate laminate veneers, while keeping the increase in pulp temperature within acceptable limits. Thinner veneers are more efficient to debond due to better energy transmission, while careful consideration of power settings is necessary to minimize risks to both pulp and enamel health.
Morford et al. (2011) [21]	Standardized IPS Empress Esthetic (EE) and IPS e.max Press HT (Emax) porcelain veneers with flat surfaces for absorption assessment.		Twenty-four extracted anterior incisor teeth prepared. Veneer thickness measured at three locations. 3M ESPE RelyX veneer cement was tested for absorption characteristics and ablation thresholds using Fourier transform infrared spectroscopy (FTIR) and visual inspection.	- FTIR spectra showed no water absorption bands in veneer materials, but the bonding cement showed a broad H_2_O/OH absorption band. - EE veneers transmitted 11.5% to 21% of laser energy, while Emax transmitted 26.5% to 43.7% - Emax transmitted roughly twice the energy at comparable thicknesses. - All EE porcelain veneers were completely removed from the tooth using the Er:YAG laser with an average removal time of 113 s.
Iseri et al. (2014) [22]	Sixty bovine mandibular incisor teeth divided into two groups (n = 30): a control group and a test group.	Er:YAG laser (VersaWave, HoyaConbio, Fremont, CA, USA) applied without water at a power of 5 W (50 Hz × 100 mJ) with a wavelength of 2940 nm	The application tip (1 mm in diameter) was positioned perpendicularly at 2 mm from the PLVs. The laser energy was applied to the test groups by scanning through the surface of the PLVs for 9 s. Scanning was performed with horizontal movements parallel to the surface.	- The control group had a significantly higher shear bond strength (27.28 ± 2.24 MPa) than the laser-irradiated group (3.44 ± 0.69 MPa). - There were statistically significant differences between the control and test groups (*p* < 0.05). - Laser energy may degrade the adhesive resin.
Zhang et al. (2018) [23]	Twelve freshly extracted teeth were prepared and bonded to veneers.	Er:YAG laser (Lite Touch, Yokne’am Illit, Israel)	Laser settings: 100 mJ and 30 Hz, theoretical fluence of 19.94 J/cm^2^. One week after bonding the veneers, Er:YAG laser with a non-contact sapphire tip and air–water spray was used for debonding at 100 mJ energy and 30 Hz frequency (Fluence 19.94 J/cm^2^). The total number of pulses was recorded at the beginning and the end of the irradiation, and by their difference, the pulses necessary to remove the veneer were calculated.	All veneers were completely and easily removed. In case of partial debonding, the remaining veneer structure was eliminated under the same conditions previously described by the authors. In case of partially debonded veneer, irradiation continued until no veneer structure was observed. Irradiation was stopped only when no veneer structure was macroscopically observed. The pulse number ranged from 17,157 to 4077 (mean range 9836). The average removal time was 328 s (standard deviation 156 s), while the removal time ranged from 136 to 572 s.
Rechmann et al. (2014) [24]	IPS E.max CAD Lithium-disilicate (LS2) (E.max CAD) and IPS E.max ZirCAD Zirconium oxide (ZrO_2_) (ZirCAD) (Ivoclar, Vivadent, Liechtenstein), either as stand-alone teeth or placed in an artificial row of teeth, were prepared to receive all-ceramic crowns.	Er:YAG (LiteTouch, Israel)	Copings and full contour crowns with either featheredge or regular margins were produced. Cement: Multilink Automix (Ivoclar). The time for Er:YAG laser debonding of each crown was then measured. Laser settings: 1100 mm diameter fiber tip with up to 600 mJ per pulse (wavelength 2940 nm, 10 Hz repetition rate, pulse duration 100 ms at 126 mJ/pulse, and 400 ms at 590 mJ/pulse). Distance: 10 mm from the crown surface following a defined pattern. Air–water spray rate: 67 mL/min.	Average debonding time for IPS E.max CAD crowns: approximately 190 s. IPS E.max ZirCAD crowns, averaging 226 s for featheredge crowns and 312 s for crowns with regular margins. E.max CAD crowns allowed for better laser energy transmission, facilitating a more effective debonding process. In contrast, ZirCAD crowns transmitted roughly 80% less laser energy, making their removal more time-consuming and challenging. The thicker walls of ZirCAD crowns necessitate higher laser energy settings to effectively reach and deteriorate the cement, particularly at contact points, which can be difficult to access.
Deeb et al. (2023) [25]	Retrospective analysis involving 29 clinical cases with a total of 52 abutments requiring the removal of various ceramic restorations using either an Er,Cr:YSGG laser (N = 6) or an Er:YAG laser (N = 46).	Er:YAG laser (LightWalker, Fotona, Slovenia) with a tipless handpiece (HO_2_, Fotona) operating at a power of 2.5–5 W; operation mode QSP/SSP; air/water spray at 2/2 or 6/6; and non-contact mode Er,Cr:YSGG laser (Waterlase, Biolase, USA) with an MX9 Turbo handpiece operating at 5W, 15 PPS; 20 air/20 water spray	The analysis evaluated the clinical procedures performed, including the type and material of the prosthetic, the type of cement used, laser setting parameters, retrieval time, and retrieval success.	Out of the 52 abutments, 50 were successfully retrieved without causing any damage (>95%)
Aksakalli et al. (2015) [26]	Thirty-nine teeth were used for shear bond strength testing (n = 13), and the remaining three teeth (one tooth for each group) were used for evaluation of the debonded bracket interface.	Group ER: Er:YAG laser (Fotona) 2 W power output at a rate of 10 Hz for 10 s. The laser irradiation of all the specimens was performed by the same operator. Laser parameters: pulse energy of 200 mJ, 2 W power, a 100-μs pulse length, pulses per second of 10 Hz, and an energy density of 25.31 J/cm^2^. Diameter of the tip was 1 mm. The levels for air and water were 90 and 80%, respectively. The laser was directed perpendicular to the porcelain surface at 1 mm. To prevent unnecessary irradiation, acrylic resin with a 4 × 6 mm hole was placed on the porcelain surface.	Group SB, sandblasting with alumina particles (50 μm); Group HFA, 9.6 % hydrofluoric acid etching. Group ER, erbium-doped yttrium–aluminum–garnet (Er:YAG) irradiation (from 1 mm distance, 2 W, 10 Hz for 10 s).	Hydrofluoric acid etching yielded the highest shear bond strength (10.8 ± 3.8 MPa). Er:YAG laser etching achieved a slightly lower bond strength (9.3 ± 1.5 MPa), but still falls within the clinically acceptable range for orthodontic bonding. Optimal bond strength for brackets to enamel is generally between 6 and 10 MPa, indicating that both methods can provide satisfactory results for orthodontic applications.
Juntavee et al. (2018) [27]	Three groups according to the surface treatment: Er-YAG laser (LE) or etching with 9.6% HF acid for 5 s (A5) or 15 s (A15).	Er-YAG laser (AT Fidelis, Fotona) through a non-contact hand-piece (R02; 1.3 mm in diameter), at the power of 200 mJ, 10 W, and 20 Hz in MSP mode (100-µs pulse length). Laser was lased perpendicular to the ceramic surface at a distance of 7 mm from the ceramic surface and in the central area of 4 × 4 mm with a water coolant for 20 s.	Machined ceramic specimens (10 × 10 × 1.5 mm) were prepared from Empress CAD (EP), and e.max CAD (EM). Ceramic veneering metal specimens (PF) were fabricated from sintered d.Sign porcelain (1.27 mm thickness) over d.Sign10 metal (0.23 mm thickness). Resin adhesive (Transbond XT) was used for attaching ceramic brackets for each group (n = 15) and cured with LED (Bluephase) for 50 s.	Er:YAG laser etching and hydrofluoric acid etching are effective methods for bonding orthodontic brackets to porcelain laminate veneers, with hydrofluoric acid providing slightly superior bond strength. However, the safety profile of Er:YAG laser etching, characterized by minimal surface damage and reduced risk to oral soft tissues, makes it a more favorable option for clinicians.
Ismatullaev et al. (2021) [28]	Occlusal surfaces of 64 caries-free human molars and vestibule surfaces of 64 first maxillary incisors were ground to obtain flat superficial dentin and flattened enamel, respectively. Four groups according to the surface etching method (37% orthophosphoric acid, Er:YAG laser–contact handpiece/scanning handpiece (1 or 2 times of scanning).	Er:YAG laser (Lightwalker, Fotona) emitting photons at a wavelength of 2.94 μm and pulse duration of 100 μs in all laser groups. The output power and repetition rate of this equipment were adjusted to be the same for all laser groups to 120 mJ and 10 Hz on enamel and dentin surfaces. The energy densities in group Er:YAG-H14 were 9.05 J/cm^2^, while in XR and XR2 groups it was calculated as 18.87 J/cm^2^.	The study was carried out in 8 groups with 15 different samples in each group, using 2 different dental tissues (enamel and dentin) and 4 different surface etching techniques. One enamel and dentin specimen representing each group was stored for SEM analysis after surface treatment procedure.	- Both laser etching and acid etching present unique advantages and challenges in enhancing bond strength in dental applications. - Effectiveness of laser etching shows promise as a viable alternative to acid etching, but can vary based on specific techniques and adhesive systems used. - By following the recommendations outlined in this report, clinicians can optimize their etching practices to achieve better bonding outcomes in dental procedures.
Oztoprak et al. (2012) [29]	Eighty freshly extracted, non-carious bovine permanent mandibular incisors, flattened and restored with lithium disilicate ceramic discs, randomly assigned to four groups (n = 20).	Er:YAG laser (VersaWave) at a power of 5 W (50 Hz × 100 mJ) with a wavelength of 2940 nm	Specimens were stored in distilled water at 37 °C for 48 h. The first group was designated as the control group, and no laser application was performed. The Er:YAG laser was applied to each specimen in the other three study groups for 3, 6, and 9 s by using the scanning method. Application tip 1 mm diameter positioned perpendicularly at a 2 mm distance from the laminate veneers. Scanning was performed with horizontal movements parallel to the surface.	Within the limits of the current study, 9 s of lasing appears to have the most favorable effect on debonding of porcelain laminate veneers.
Suliman et al. (2024) [30]	Four groups of 10 crowns, prepared and tested for laser-assisted removal, based on the type of ceramic material and yttria content: - 3 mol% Y-TZP zirconia (KATANA Zirconia High Translucent, Kuraray Noritake, Tokyo, Japan); - 4 mol% Y-TZP zirconia (KATANA Zirconia Super Translucent Multi Layered, Kuraray Noritake); - 5 mol% Y-TZP zirconia (KATANA Zirconia High Translucent, Kuraray Noritake) - Lithium disilicate (control group).	Laser Wavelength: 2940 nm Power Settings: 335 mJ, 15 Hz, 5.0 W Mode: Super short pulse (SSP) Handpiece: Tipless, with water and air spray Distance: Maintain 5–8 mm from the crown surface Cooling: Continuous water and air spray during application	Forty extracted human premolars were cleaned and embedded in acrylic resin, with crowns prepared using high-speed rotary instruments under water cooling. Crowns made of zirconia with varying yttria contents (3%, 4%, 5% mol%) and lithium disilicate were used, with their surfaces examined via SEM to assess damage post-irradiation. The laser was applied with continuous motion across all crown surfaces (buccal, lingual, mesial, distal, occlusal) until crown dislodgement, with darkening of cement indicating ablation and adhesive disruption. The ceramic surfaces were examined under SEM for structural damage, residual cement, and smear layers. The dentin surfaces were also inspected for damage, residual cement, and smear layers, with no significant structural damage observed.	The Er:YAG laser efficiently enabled the extraction of diverse ceramic crowns, including zirconia with varying yttria concentrations (3%, 4%, 5% mol%) and lithium disilicate, while exhibiting minimal structural damage to both ceramic and dental surfaces. The employed laser settings were enough for crown dislodgement, rendering the procedure safe and less intrusive than conventional rotational techniques. The retrieval time fluctuated based on the yttria concentration in zirconia, with elevated yttria levels (5%) leading to reduced debonding durations, suggesting that yttria content affects laser effectiveness.
Zhang et al. (2024) [31]	Control group: No Er:YAG laser debonding treatment (n = 10). 4 W laser group: Laser set to 400 mJ energy, 10 Hz frequency, with specific parameters, treated for 300 s. 5 W laser group: Laser set to 500 mJ energy, 10 Hz frequency, treated similarly. 6 W laser group: Laser set to 600 mJ energy, 10 Hz frequency, treated similarly.	Power range: 4 W to 6 W, Energy density approximately between 37.69 J/cm^2^ and 45.23 J/cm^2^. QSP (Q-switched pulse) mode, 10 Hz frequency, 500 mJ pulses at 4 W or 600 mJ at 6 W. Water spray at 0/8 and air spray at 6/8. Controlled, sweeping motion over the restoration margins and internal surfaces.	One hundred and sixty specimens (25 mm × 8 mm × 1.5 mm), four types of zirconia ceramics: self-glazed zirconia (SGZ), 3Y-TZP, 4Y-PSZ, and 5Y-PSZ, prepared using CAD/CAM technology, sintered, glazed, and ultrasonically cleaned. Divided into four groups: control (no laser), and three laser groups with different power settings (4 W, 5 W, 6 W). Laser parameters included a fixed frequency of 10 Hz and a treatment duration of 300 s for each laser group [2]. Optical properties (color difference ΔE and transparency parameter TP) were measured before and after laser treatment [2]. Mechanical properties were evaluated via flexural strength tests according to ISO 6872 [32] standards, using a universal testing machine with a 15 mm span and 1 mm/min crosshead speed until failure [4]. SEM analysis was performed to observe surface morphology and detect any microcracks or damage post-treatment.	The Er:YAG laser debonding did not significantly affect the optical or mechanical properties of the examined zirconia ceramics, including the innovative 5Y-PSZ, 4Y-PSZ, 3Y-TZP, and SGZ. No indications of heat or photoablation effects were seen; surface roughness and translucency characteristics were predominantly unchanged, with minor reductions in translucency detected at elevated laser energy. Er:YAG laser debonding is a secure and efficient technique for the removal of zirconia restorations, preserving their structural integrity and cosmetic qualities, hence facilitating the possible reusability of zirconia restorations in clinical settings.
Jiang et al. (2024) [33]	Groups based on the type of zirconia (3Y-TZP and 5Y-TZP) and specific laser settings, including laser energies (80 mJ to 260 mJ) and frequencies (10 Hz and 20 Hz). Each subgroup consisted of five specimens, allowing for comparison of debonding times, temperature changes, and surface characteristics for different combinations of zirconia types and laser parameters.	Er:YAG laser, non-contact mode, 90° angle, 1 mm distance from the zirconia surface. Laser settings - Energy levels 80 mJ to 240 mJ. - Groups formed by the combination of frequency and energy (F1E8, F2E8, F1E10, F2E10). “F” stands for frequency (10 Hz or 20 Hz) and “E” for energy in millijoules. - Laser applied uniformly in a stacked tile shape according to the specified output settings.	Extracted, caries-free teeth were collected, prepared by removing soft tissues, and stored in distilled water at 4 °C for less than one month. Zirconia specimens were fabricated via CAD/CAM technology, divided into two groups based on material type (3Y-TZP and 5Y-TZP), and bonded to dentin blocks prepared from extracted molars. Specimens randomly assigned to laser treatment groups. Data collection included debonding time, dentin temperature changes, surface characteristics, surface roughness via a contact profilometer, and flexural strength using a universal testing machine	Results: The debonding time varied from 4.8 to 160.4 s. The laser parameters influenced both the efficiency and safety of the debonding operation. Conclusions: The results indicate that Er:YAG laser settings substantially affect debonding efficiency and safety, necessitating optimal conditions for successful zirconia removal without harming adjacent tissues.
Suliman et al. (2024) [34]	G1a: Zirconia crowns, air-particle abrasion, bonded with Panavia V5 resin cement, primer application. G1b: Zirconia crowns, self-adhesive bonding agent (Scotchbond Universal) without air-particle abrasion. G2a: Lithium disilicate crowns, hydrofluoric acid, bonded with RelyX Ultimate resin cement. G2b: Lithium disilicate crowns, Scotchbond Universal, without hydrofluoric acid treatment.	Er:YAG laser, 2.94 μm wavelength. Power setting: 5 W, pulse duration of 50 ms, frequency of 15 Hz. Water/air spray of approximately 4/4. Tip-to-crown distance: 5–8 mm, continuous axial motion.	CAD/CAM crowns, zirconia, or lithium disilicate blocks. Crowns were seated on prepared teeth with manual pressure, excess cement was removed after tack curing, and final curing was performed with a curing light. Debonding was performed using Er:YAG laser irradiation at 2940 nm with parameters: 335 mJ, 15 Hz, 5.0 W, with water/air spray, directed perpendicular to the crown, until crowns could be removed by gentle tapping or digital manipulation. During laser irradiation, signs of cement ablation and disruption of the adhesive seal were observed, facilitating crown removal.	Results: The mean debonding times were 5.75 ± 2.00 min for zirconia crowns bonded with 2-bottle adhesive resin cement (G1a), 4.79 ± 1.20 min for zirconia crowns using 1-bottle cement (G1b), 1.69 ± 0.49 min for lithium disilicate crowns subjected to hydrofluoric acid treatment (G2a), and 1.12 ± 0.17 min for lithium disilicate crowns without hydrofluoric acid (G2b). No notable variations were detected between the 2-bottle and 1-bottle cement groups of the same ceramic type; however, substantial differences were identified between the zirconia and lithium disilicate groups, with lithium disilicate crowns exhibiting a quicker debonding rate. Conclusions: Er:YAG laser irradiation is a secure and effective technique for debonding zirconia and lithium disilicate crowns, exhibiting no substantial variation in debonding durations between the two adhesive systems evaluated.

TT: tooth treatment. 3STE: 3-step total-etch adhesive system (etching: 37% orthophosphoric acid gel (Total Etch; Ivoclar Vivadent, Liechtenstein) for 15 s; rinsing and drying. Universal adhesive (Adhese Universal; Ivoclar Vivadent, Liechtenstein) was homogeneously applied to the etched surface for 20 s, air-thinned for 20 s with air spray and light-cured for 10 s, as instructed by the manufacturer). HF: hydrofluoric acid.

**Table 4:** Specimen randomization, control group, standardized specimens, manufacturer’s instructions, single operator, availability of outcome data, and overall assessment for the articles on removal of veneers using Er:YAG laser (references [11,12,13,14,15,17,18,19,20,21,22,24,25,26,27]).

Study	Specimen Randomization	Control Group	Standardized Specimens	Manufacturer’s Instructions	Single Operator	Availability of Outcome Data	Overall Assessment
Luong & Shayegan (2018) [14]	−	+	+	+	−	+	++++ − −
Almeida et al. (2009) [15]	+	+	+	+	−	+	+++++ −
Dostalova et al. (2016) [16]	−	+	+	+	−	−	+++ − − −
Gozneli et al. (2023) [17]	−	−	+	+	−	+	+++ − − −
ALBalkhi et al. (2018) [18]	+	−	+	+	−	+	++++ − −
Karagoz-Yldirak & Gozneli (2020) [19]	+	−	+	+	−	+	++++ − −
El-Damanhoury et al. (2022) [20]	−	−	+	+	−	+	+++ − − −
Morford et al. (2011) [21]	−	−	+	+	−	+	+++ − − −
Iseri et al. (2014) [22]	+	+	+	+	+	−	+++++ −
Zhang et al. (2018) [23]	−	−	+	+	+	+	++++ − −
Rechmann et al. (2014) [24]	−	−	+	+	−	−	++ − − − −
Aksakalli et al. (2015) [26]	+	−	+	+	+	+	+++++ −
Juntavee et al. (2018) [27]	+	−	+	+	−	+	++++ − −
Ismatullaev et al. (2021) [28]	−	+	+	+	−	−	+++ − − −
Oztoprak et al. (2012) [29]	+	+	+	+	−	−	++++ − −
Suliman et at (2024) [30]	+	+	+	+	−	−	+++++ −
Zhang et al. (2024) [31]	−	+	+	+	−	+	++++ − −
Jiang et al. (2024) [33]	+	+	+	+	−	−	++++ − −
Suliman et al. (2024) [34]	+	+	+	+	+	−	+++++ −

## Data Availability

Data are contained within the article.
